# Physicians’ Diary for 1857

**Published:** 1856-12

**Authors:** 


*Physicians' Diary for* 1857.—This little daily reminder of duty, is now ready for orders. In its appearance it exceeds our expectation. It is only one-half the weight of the "Medical Record," published last year, and is even lighter than the "Physicians' Visiting List." It furnishes abundant space for the record of names, providing for thirty patients per week, with opportunity for record, on the opposite page; of the age of the patient, name of the disease, termination, and brief remarks. Orders enclosing 75 cents, may be addressed to Phinney & Co., Buffalo, and the Diary will be mailed pre-paid.

